# Editors’ Book Table

**Published:** 1873-06

**Authors:** 


### Editors' Book Table.

[NOTE. — All works reviewed in the columns of the CHICAGO MEDICAL JOURNAL may be found in the extensive stock of W. B. KEEN, COOKE & CO., whose catalogue of Medical Books will be sent to any address upon request.]

BOOKS RECEIVED.

*Hand Book for the Physiological Laboratory.* By E. KLEIN, M.D., Assist. Prof. in the Pathological Laboratory of the Brown Institution, London—formerly Privat-docent in Histology in the University of Vienna; J. BURDON-SANDERSON, M.D., F.R.S., Prof. of Practical Physiology in University College, London; MICHAEL FOSTER, M.A., M.D., F.R.S., Fellow of and Prælector of Physiology in Trinity College, Cambridge; and T. LANDER BRUNTON, M.D., D.Sc., Lecturer on Materia Medica in the Medical College of St. Bartholomew's Hospital, London. Edited by J. BURDON-SANDERSON. In Two Vols. With 133 Plates, containing 353 Illustrations. Vol. I, Text; Vol. II, Plates. Philadelphia: Lindsay & Blakiston. 1873. 8vo. Text (and Index) 585 pages.

This is a most superb work, and fills a hiatus which every physiological student has lamented. Under its guidance all the more important experiments whereby the great science of physiology has been advanced, and rational therapeutics suggested, may be verified, and skill in manipulation and method gained for the initiation of new and, perchance, bolder inquiries. For, doubt it who may, the sure basis of medicine, as either science or art, is only to be found in the minute and exact experimentation which modern physiology demands, supplemented by the wider, more comprehensive, more dazzling and striking generalizations of modern meteorology and its kindred sciences.

*A Hand Book of Medical Electricity.* By HERBERT TIBBITS, M.D., L.R.C.P., London, Medical Superintendent of the National Hospital for the Paralyzed and Epileptic; Medical Officer for Electrical Treatment to the Hospital for Sick Children, Great Ormond St. With Sixty-four Illustrations. Philadelphia: Lindsay & Blakiston. 1873. 8vo. Cloth. Pp. 164.

The four chapters of this concise and useful manual discuss respectively: medical electricity and electro-medical instruments; the application of electricity; electricity as an aid to diagnosis; and electro-therapeutics.

*Mechanism of the Ossicles of the Ear and Membrana Tympani.* By H. HELMHOLTZ, M.D., Professor of Physiology in the University of Berlin, Prussia. Translated from the German, with the author's permission, by ALBERT H. BUCK and NORMAND SMITH, of New York; with Twelve Illustrations. New York: William Wood & Co., 27 Great Jones Street. 1873. Thin 8vo. Cloth. Pp. 69.

Probably the most complete and thorough exposition of the subject extant. The mathematical relations are developed with extreme care and accuracy, with an abstruseness, indeed, exceedingly perplexing to the ordinary scholar, nevertheless the work in great part is readily comprehensible and instructive.

*A Treatise on the Principles and Practice of Medicine; Designed for the Use of Practitioners and Students of Medicine.* By AUSTIN FLINT, M.D., Professor of the Principles and Practice of Medicine and Clinical Medicine, in the Bellevue Hospital Medical College. Fourth Edition, carefully Revised. Philadelphia: Henry C. Lea. 1873. 8vo. Pp. 1070.

It is unnecessary, of course, to either introduce or eulogize this now standard treatise. All the colleges recommend it as a text book, and there are few libraries in which one of its editions is not to be found. It has accomplished a great work, if in nothing else, in causing the abandonment of the hypermedication that most of its predecessors encouraged and advised. Briefly, it is replete with conservative medicine.

The present edition has been thoroughly revised so as to bring it up to the author's present level of experience and reading. His own clinical studies, and the latest contributions to medical literature, both in this country and in Europe, have received careful attention, so that some portions have been entirely rewritten and about seventy pages of new matter have been added. The additions, as might have been expected, are especially to the section devoted to diseases of the nervous system.

PAMPHLETS, ETC.

*The Obstetrical Journal of Great Britain and Ireland, Including Midwifery and the Diseases of Women and Children.* Edited by JAMES H. AVELING, M.D., Physician in the Chelsea Hospital for Women, and ALFRED WILTSHIRE, M.D.,Ass't Physician Accoucheur to St. Mary's Hospital; to which is added an American Supplement. Edited by WILLIAM F. JENKS, M.D., Obstetric Physician to the Philadelphia Dispensary. To appear in Monthly Numbers, of about Eighty octavo pages each, very handsomely printed, price $5 per annum, in advance. Single Numbers, 50 cents each.

The first (April) number of this new journal has reached our table, and seems to fully merit the patronage asked for it. Among the contributors we recognize a large number of names of world-wide reputation. The American Supplement is to be separately paged for convenience of reference. Address Henry C. Lea, Philadelphia.

*Ophthalmic and Aural Surgery Reports.* By JULIAN J. CHISHOLM, M.D., etc., etc. Baltimore, Md.

*Quarterly Summary of the Transactions of the College of Physicians of Philadelphia*, from Feb. 7, 1872, to Oct. 16, 1872, inclusive.

*On Ovariotomy.* By J. MARION SIMMS, M.D., one of the Surgeons of the New York State Woman's Hospital, etc. Pp. 85.

The feature of this monograph is, we may say, developed in these sentences:

"The time will assuredly arrive when peritonitis, so called, will not kill, because we will learn that the effusions in the peritoneal may be as safely evacuated as those of the pleural cavity; that the danger will consist, not in opening the peritoneal cavity, but in keeping it closed with its retained fluids to poison the blood and take the life of the poor sufferer. The time will also come, when gunshot and other wounds of the abdomen, and perforations of the intestine, will be treated by opening the peritoneal cavity, and washing out or draining off the septic fluids that would otherwise poison the blood; for death in all these cases is produced by the same causes and in precisely the same way, and they will require the same plan of treatment."

All of which we steadfastly believe.—The author will accept our thanks for this valuable and interesting pamphlet.

*The Function of the Eustachian Tube in its Relation to the Renewal and Density of the Air in the Tympanic Cavity, and to the Concavity of the Membrana Tympani.* By THOS. F. RUMBOLD, M.D., St. Louis. Pp. 40.

*Meteorology and its Professors.* A review by the Editor of the Canada Med. and Surg. Journal, Counter Statement, etc., etc. By THOS. D. KING, Montreal. 1873. Pp. 26.

*Transactions of the Medical Society of the State of California,* during the years 1871–72. Sacramento. 1872. Pp. 228.

